# Potential of Sulforaphane as a Natural Immune System Enhancer: A Review

**DOI:** 10.3390/molecules26030752

**Published:** 2021-02-01

**Authors:** Andrea Mahn, Antonio Castillo

**Affiliations:** 1 Departamento de Ingeniería Química, Facultad de Ingeniería, Universidad de Santiago de Chile (USACH), Santiago 8330111, Chile; 2 Departamento de Biología, Facultad de Química y Biología, Universidad de Santiago de Chile (USACH), Santiago 8330111, Chile; antonio.castillo@usach.cl

**Keywords:** sulforaphane, immunological response, cellular mechanism

## Abstract

Brassicaceae are an outstanding source of bioactive compounds such as ascorbic acid, polyphenols, essential minerals, isothiocyanates and their precursors, glucosinolates (GSL). Recently, GSL gained great attention because of the health promoting properties of their hydrolysis products: isothiocyanates. Among them, sulforaphane (SFN) became the most attractive one owing to its remarkable health-promoting properties. SFN may prevent different types of cancer and has the ability to improve hypertensive states, to prevent type 2 diabetes–induced cardiomyopathy, and to protect against gastric ulcer. SFN may also help in schizophrenia treatment, and recently it was proposed that SFN has potential to help those who struggle with obesity. The mechanism underlying the health-promoting effect of SFN relates to its indirect action at cellular level by inducing antioxidant and Phase II detoxifying enzymes through the activation of transcription nuclear factor (erythroid-derived 2)-like (Nrf2). The effect of SFN on immune response is generating scientific interest, because of its bioavailability, which is much higher than other phytochemicals, and its capacity to induce Nrf2 target genes. Clinical trials suggest that sulforaphane produces favorable results in cases where pharmaceutical products fail. This article provides a revision about the relationship between sulforaphane and immune response in different diseases. Special attention is given to clinical trials related with immune system disorders.

## 1. Introduction

Many of the current synthetic drugs come from natural products of plant origin. Even some plant-derived bioactive compounds have been proposed as possible therapeutic solutions to fight highly prevalent diseases such as cancer [1]. 

Sulforaphane (SFN), an isothiocyanate (ITC) widely distributed in Brassicaceae plants, has generated great interest in the last 15 years, with an exponentially growing number of scientific articles reaching around 250 in 2020 and a total of 2315 since 1948 (PubMed, https://pubmed.ncbi.nlm.nih.gov/, last access 21 December 2020). This is due to the outstanding health promoting properties of SFN which are related with its high capacity to induce Phase II detoxifying enzymes, being 14-fold higher than other potent phytochemicals such as quercetin. Additionally, SFN exhibits the highest bioavailability among well-known antioxidant phytochemicals, such as quercetin (20-fold higher) [2] and curcumin (80-fold higher) [3]. This confers SFN a high potential to be used as nutraceutical to improve health status or as pharmaceutical to treat some disease states. 

**Figure 1:**
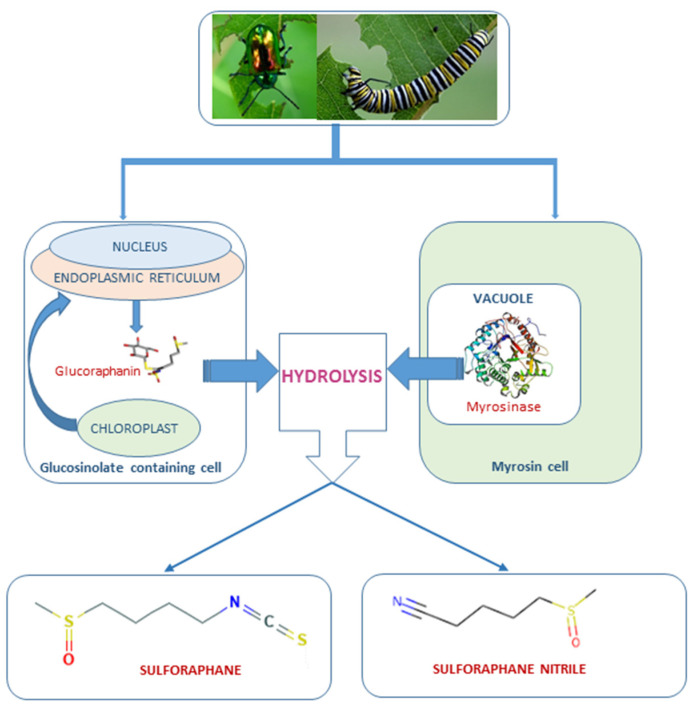
Myrosinase—glucoraphanin system in Brassicaceae plants. Glucosinolates are located in specialized glucosinolate-containing cells, while myrosinase is stored in the vacuoles of the myrosin cells. After mechanical disruption of plant tissue, the substrate and enzyme come in contact and the hydrolysis occurs, resulting in different products, among which it is found sulforaphane [7].

**Figure 2:**
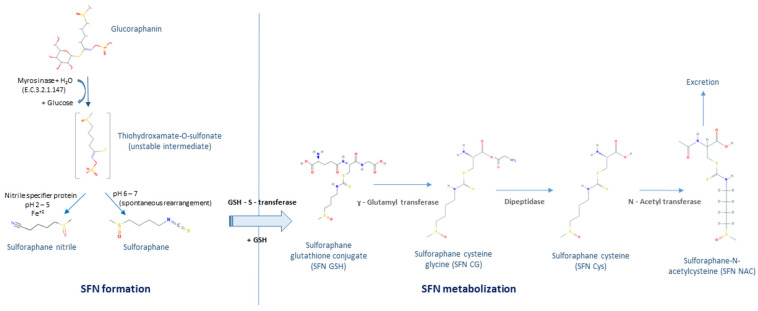
Formation and metabolization of sulforaphane. Sulforaphane (SFN) is formed by the hydrolysis of glucoraphanin catalyzed by either plant or bacterial myrosinase. After intake, SFN is metabolized through the mercapturic acid pathway. Initially, isothiocyanates are conjugated with glutathione (GSH) in a glutathione transferase (GST)-catalyzed reaction. Then, successive cleavage reactions catalyzed by γ-glutamyltranspeptidase, cysteinylglycinase, and N-acetyltransferase occur to generate sulforaphane-N-acetylcysteine (SFR-NAC) [8].

SFN comes from the enzymatic hydrolysis of glucoraphanin, a glucosinolate stored as inactive precursor in the plant cells. The hydrolysis of glucoraphanin occurs through myrosinase (E.C. 3.2.1.147), which is compartmentalized in the vegetable inside myrosin cells. The reaction proceeds after tissue disruption that can be produced by insects and herbivores attack or by processing and chewing the vegetable [4]. The products of the hydrolysis reaction vary depending on the chemical conditions where the reaction occurs. The myrosinase—glucosinolate system belongs to the defense system of the plant and therefore some of the products that come from glucosinolates hydrolysis are toxic [5]. Figure 1 depicts the myrosinase—glucosinolate (glucoraphanin) system in plants. In mammals, SFN can be administered directly in its active form or as glucoraphanin which undergoes the hydrolysis during digestion by the action of vegetable and gut microflora myrosinases. After intake, SFN follows the mercapturic acid pathway until its conversion in dithiocarbamates and is finally excreted [6]. Figure 2 shows the formation and metabolic degradation of SFN.

Several efforts have been conducted in order to exploit the health-promoting effects of SFN on humans. Its direct administration has been limited because of the instability of SFN. Some research about SFN stabilization is being conducted [9]. Another way to administer SFN to humans is through broccoli sprout extracts or minimally processed broccoli. Some processing conditions that maximize SFN content in processed broccoli have been reported [10,11,12,13,14], resulting in an important amount of information about the processing conditions that achieve this goal. Given the instability of SFN and the possibility to maximize SFN in processed vegetable, most clinical studies about the effect of SFN use broccoli extracts or powder, and focused on validating the efficacy of SFN-rich food, not in SFN as a drug.

The first report about the effect of sulforaphane on health dates from 1992, when Zhang et al. [15] suggested that SFN was a potent activator of cellular defense systems. Later Bonnesen et al. [16] informed that SFN and other isothiocyanates showed a preventive effect on colon tumorigenesis since these compounds stimulate apoptosis and enhance cell defense against molecules that produce gene toxicity. Since then, several in vitro and in vivo studies about the effect of SFN have been conducted, including clinical trials, resulting in relevant information regarding prevention and treatment of diseases such as pancreatic cancer [17], breast cancer [18], diffuse axonal injury [19], lymphomas [20], liver cancer [21], leukemia [22], and prostate cancer [23]. Moreover, SFN has cardio protective properties [24], the ability to prevent aging and neurodegeneration [25], and to protect from gastric ulcer [26]. Additionally, several clinical trials are currently in progress or already finished. 

This review aims at presenting the most recent advances of research about the effects of SFN on the immune system, considering in vitro studies, which were performed using animal or human cells in culture, and in vivo studies, which were animal or human clinical intervention trials.

## 2. Mechanisms of Action of SFN on Immune System

Sulforaphane exerts a pleiotropic effect on immunological response. The mechanism is based on activation of nuclear factor (erythroid-derived 2)-like (Nrf2) which triggers cellular defense mechanisms. There is induction of Phase II detoxifying enzymes as well as antioxidant enzymes, and down regulation of Phase I enzymes by inactivation of NFκβ. The final effect of SFN varies with cell type. In T-cells, the response to SFN exposure is the generation of a pro-oxidant environment, with an increase of intracellular reactive oxygen species (ROS) and a decrease in intracellular glutathione (GSH) levels, that produces a block of the T-cell-mediated immune response. SFN is able to create a pro-oxidative ROS enriched milieu in primary human T-cells. It inhibits co-stimulation initiated T-cell activation and proliferation by depletion of GSH and oxidation of proteins at redox active cysteine residues. Importantly, SFN also enhances the ROS levels in lymphocytes within whole blood of RA (rheumatoid arthritis) patients and inhibited the production of pro-inflammatory TH17 related cytokines. This immunosuppressive effect of SFN on T-cells can be desirable in autoimmune or inflammatory diseases, but it would be detrimental in other chronic diseases such as cancer since the T-cell-mediated immune response is important for immune surveillance of tumors. Therefore, caution should be exercised, as SFN could interfere with the successful application of immunotherapy by immune checkpoint inhibitors (e.g., CTLA-4 antibodies and PD-1/PD-L1 antibodies) or CAR (chimeric antigen receptors) T-cells in cancer patients, and a combination of both treatments could not be advisable [27]. 

Although there is a good amount of evidence that indicates that SFN is a potent anticancer compound and that its main mechanism of action would occur through the activation of Nrf2, recent publications present controversial results that indicate that the activation of Nrf2 contributes to the whole process of pathogenesis, promotes cancer progression and metastasis while conferring resistance to chemo- and radiotherapy, and has a poor prognosis, a phenomenon that has been described as the “dark side” of Nrf2 [28]. Therefore, in accordance with the above, Nrf2 could be a promising target in cancer therapy. However, research related to Nrf2 inhibitors is still incipient [29].

**Figure 3:**
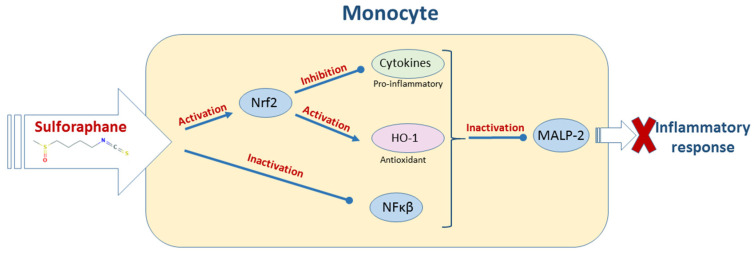
Mechanism of inflammatory response suppression induced by sulforaphane in bacteria-infected monocytes.

In monocytes and macrophages, SFN inhibits pro-inflammatory cytokines and activates antioxidant enzymes through Nrf2 modulation, resulting in an anti-inflammatory effect (Figure 3) useful for the treatment of bacterial and viral related diseases. SFN is widely recognized as among the most powerful natural anti-cancer agents, but its mechanism of action is not fully understood so far. This owes to the multi-factorial nature of this disease and to the pleiotropic effect of SFN. However there is evidence that supports SFN to exert an antioxidant effect in tumor cells [30]. The mechanisms that underlay SFN effect on immunological system in different diseases are presented below. 

### 2.1. Autoimmune/Inflamatory Diseases

SFN exerts its effect on immune system through different biochemical and cellular mechanisms, among them the downregulation of pro-inflammatory cytokines, T-cells suppressing, and activation of adenosine monophosphate activated protein kinase (AMPK) signaling pathway. Even though these processes have a suppressive effect, this is desirable in cases of autoimmune/inflammatory diseases. 

Townsend and Johnson [31] studied the effect of sulforaphane on pro-inflammatory markers and target genes of nuclear factor erythroid 2 (NFE2)—related factor 2 (Nrf2) in mice subjected to lipopolysaccharide (LPS) challenge. They found that SFN decreased pro-inflammatory markers such as interleukin 1-β (IL-1β) and interleukin 6 (IL6) as response to LPS-treatment. The authors propose that the anti-inflammatory effect of SFN was regulated by the Nrf2 pathway. 

Deng et al. [32] demonstrated that SFN delivered as broccoli nanoparticles to mice is involved in prevention of colitis, an autoimmune disease that can lead to ulcers. The mechanism consists in the induction of tolerogenic dendritic cells by adenosine monophosphate activated protein kinase (AMPK), thus regulating the intestinal immune homeostasis. Accordingly, SFN could have preventive or therapeutic application on some intestinal inflammatory diseases due to its activating effect of AMPK signaling pathway. 

Liang et al. [33] studied the effect of sulforaphane on the redox regulation in human T-cells, in order to uncover the mechanism that underlays the immunosuppressive effect of SFN in chronic Th17-related diseases, such as rheumatoid arthritis. They reported that SFN exerts a redox-related immunosuppressive effect on untransformed human T-cells, downregulation of the pro-inflammatory Th17 cytokines associated with autoimmune/inflammatory diseases (IL-17A, IL-17F and IL-22), and inhibition of cartilage-disruptive proteins. These processes produce a significant reduction in the clinical symptoms. Since this study was conducted ex vivo, the results cannot be extrapolated to the effect in humans. 

Some authors investigated the effect of sulforaphane on immune-associated inflammatory diseases of the central nervous system (CNS), such as Alzheimer and Parkinson, concluding that SFN has anti-inflammatory and anti-oxidant effect [34,35]. The mechanism that underlies this kind of disease relies on promotion of leukocyte traffic across the blood-brain barrier by the action of reactive oxygen species (ROS) [36]. ROS induce myelin breakdown and neuronal injury, among other effects. Additionally, the infiltrated cells increase the production of ROS, thus contributing to the advance of the CNS diseases [37]. Yoo et al. [38] administered SFN (orally, 50 mg/kg/day over 14 days) to an autoimmune encephalomyelitis model (mouse). The clinical symptoms of SFN–treated animals were diminished significantly in comparison with those observed in control animals. This was attributed to the anti-inflammatory and anti-oxidative effects of SFN, resulting in neuroprotection. Accordingly, SFN seems a promising alternative to traditional drugs, which are expensive and most importantly have undesirable side-effects. 

### 2.2. Pulmonary Diseases

Information about the effect of SFN on immune system in lung diseases is poorly documented so far. Recently, Patel et al. [39] presented evidence that SFN can act as prophylactic in hyperoxia-induced lung injury or hyperoxia-compromised macrophage function in phagocytosis. The results presented in this study suggest that SFN can alleviate hyperoxia-induced inflammatory acute lung injury by increasing macrophage phagocytosis via inhibiting the accumulation of extracellular HMGB1 (high-mobility group box 1 protein). Thus, by reducing the toxic effects of extracellular HMGB1, it is possible to maintain the functions of pulmonary macrophages and the integrity of lung tissues under oxidative stress. This is the first report in which is shown that SFN attenuate hyperoxia-induced macrophage dysfunction through an HMGB1-mediated pathway. The authors concluded that the supplementation of SFN during oxygen therapy may prevent lung damage and preserve lung cell functions and lung tissue integrity, thus providing a promising therapeutic approach for patients receiving mechanical ventilation.

### 2.3. Viral Diseases

Literature about the effect of SFN on immunological system during viral infection is scarce. There are studies showing that SFN may help an organism to fight against some types of virus, mainly HIV, influenza, hepatitis C, and most recently COVID-19. These studies suggest that SFN acts by restoring the immune system and downregulating free radicals production, mediated through modulation of antioxidant genes expression by the transcription factor Nrf2. 

Jin-Nyoung et al. [40] studied the effect of administering isothiocyanates (benzyl isothiocyanate, indolo[3,2-b]carbazole, indole-3-carbinol, phenethyl isothiocyanate, and sulforaphane) on the life span of leukemia retrovirus infected-mice. The authors reported that mice treated with benzyl isothiocyanate, phenethyl isothiocyanate, or sulforaphane significantly extended their life span in comparison with the control retrovirus-infected group. Accordingly, those three ITC retarded the evolution of the infection with LP-BM5 retrovirus to murine AIDS. Furuya et al. [41] investigated the effect of SFN on human macrophages and T-cells after infection with HIV. The authors demonstrated that, unlike other viruses like Dengue virus (DENV) or Marburg virus (MARV) that benefit from Nrf2, HIV infection is blocked with the activation of Nrf2 in primary macrophages. This effect was not detected in T-cells. SFN modulates Nrf2 and results in reprogramming gene expression in macrophages. Finally, it was proposed that SFN is capable to induce an antiviral response in human macrophages against HIV, arising as a promising therapy.

In contrast to the effect of Nrf2 on the HIV infection, the oxidative stress generation during DENV infection stimulates the transcription factor Nrf2, which tightly regulates ROS levels as well as innate immune and apoptotic responses to DENV infection, limiting both antiviral and cell death responses to the virus by feedback modulation of oxidative stress. Confirming the above, silencing of Nrf2 by RNA interference increased DENV-associated immune and apoptotic responses [42]. On the other hand, MARV directly increases Nrf2 levels through a protein called VP24. This protein, like SFN, interacts with Keap1 (Kelch-like ECH-associated protein 1), a negative regulator of Nrf2. Binding of VP24 to Keap1 Kelch domain releases Nrf2 from Keap1-mediated inhibition, promoting persistent activation of diverse cytoprotective genes implicated in cellular responses to oxidative stress and regulation of inflammatory responses. The authors demonstrated that there is increased expression of Nrf2-dependent genes both during MARV infection and upon transient expression of MARV VP24. Finally, Nrf2-deficient (Nrf2^-/-^) mice can control MARV infection when compared to lethal infection in wild-type animals, indicating that Nrf2 is critical for MARV infection [43]. 

Hepatitis C Virus (HCV) is susceptible to heme-oxigenase-1 (HO-1) which interferes with the replication of viruses like HIV and Hepatitis B [44,45]. Since SFN is a potent activator of phase II antioxidant enzymes, like HO-1, Yu et al. [46] studied the effect of SFN on Huh-7 cells infected with HCV. The authors demonstrated that SFN suppresses replication of HCV by inducing HO-1 expression through activation of Nrf2 pathway. 

Efforts have been made to elucidate the role of SFN in immunological response to influenza. Some phytochemicals have shown to enhance immunological response against influenza, such as glucans [47] and sulforaphane [48], the latter associated to Nrf2 expression that blocks influenza A entry and replication in human nasal epithelial cells. Vaclav and Jana [49] investigated the effect of a glucan–SFN combination on influenza in a mouse model. They evaluated immunological response by assessing some immune reactions, virus concentration, and animal survival. The results suggested that both phytochemicals had a synergistic effect on stimulation of immunological system. Müller et al. [50] conducted a clinical trial to evaluate the effect of SFN-rich broccoli sprouts homogenate on peripheral blood mononuclear cells (PBMC) after administering a nasal vaccine dose of live attenuated influenza virus (LAIV) to healthy subjects. They found significant differences between the response to BSH (broccoli sprout homogenates) and placebo, observing that LAIV significantly reduced NKT (natural killer T) and T-cell populations. The authors conclude that nasal influenza infection may induce complex changes in peripheral blood NK cell activation, and that BSH (rich in SFN) effect may be important for enhanced antiviral defense responses. Li et al. [51] studied the effect of SFN on influenza A virus replication in Madin-Darby canine kidney cells. They detected an increased accumulation of Nrf2 factor triggered by SFN, resulting in a decrease of virus replication. 

During the last year, the world has been shocked by the abrupt irruption of COVID-19 and the scientific community has been devoted to find insights that help fight against this disease. A way to reduce the severity and mortality generated by acute respiratory distress syndrome (ARDS) produced by SARS-COV 2 is to strengthen the immune system. ARDS produces a dysregulation of the immunological system, and in the most severe cases, the release of pro-inflammatory cytokines and loss of T-cells in the infected organism [52]. There is evidence of the antiviral effect of Nrf2 on respiratory syncytial virus infection [53] and on SARS-COV 1 [54]. Based on information about viruses that belong to the same family, it has been proposed that compounds that activate Nrf2 could probably help to diminish these effects. Cuadrado et al. [55] suggested that due to its ability to activate Nrf2, induce antioxidant enzymes, reduce pro-inflammatory cytokines, and its efficacy and safety, SFN is a promising candidate to counteract inflammatory reaction and protect lungs from severe damage during SARS COV 2 infection. Finally, Horowitz and Freeman [56] suggested that clinical trials including administration of Nrf2-activating molecules, such as SFN, are imperative to support a possible three-party strategy to fight the COVID-19 pandemic, which includes prevention, diagnostic, and treatment. 

### 2.4. Bacterial Diseases 

Research about the effect of SFN on immunological system during bacterial infections is incipient. Currently there are reports that consider *H. pylori*, *S. aureus*, *E. coli* and *M. pneumoniae*. Although SFN exhibits direct bactericidal activity, it triggers an immunological response to *H. pylori* infection in the stomach mucosa. SFN acts by activating Nrf2 and downregulating NF-κB, whose joint action modulates antioxidant and anti-inflammatory response in the host [57,58]. As a consequence, SFN exerts a protective effect from gastritis and gastric ulcer. Yanaka [59] conducted in vitro and in vivo studies about the effect of SFN on *H. pylori* infection. The outcomes demonstrated that SFN significantly reduced the bacterium viability and alleviated gastritis in animal models and in humans. 

Haodang et al. [60] studied the response of monocytes stimulated with *Mycoplasma pneumoniae* lipopeptide to SFN exposure. Pathological injury of *M. pneumoniae* in lungs relates with inflammation that stimulates immune response of the host triggered by lipid polysaccharide (LPS) excretion by the bacteria. The authors found that SFN inhibited the expression of pro-inflammatory cytokines and activated the expression of HO-1 after the induction of Nrf2. As a result, SFN reduced lung inflammation in an animal model. The mechanism proposed by [60] is depicted in Figure 3. 

Ali et al. [61] investigated the effect of four Nrf2 activators on bacteria-infected macrophages, among them, SFN. Macrophages were infected either with *E. coli* or *S. aureus* and the intracellular viability of bacteria was evaluated. SFN significantly reduced intracellular bacteria survival in PBMC-derived macrophages. Even though the authors do not present any mechanism, they propose that the intra and extra cellular bactericidal effect of SFN relies on the anti-inflammatory and antioxidant milieu produced inside the macrophages. SFN, as a potent Nrf2 activator, seems a promising therapeutic option for Gram (+) and Gram (−) bacterial infections since it modulates antioxidant and anti-inflammatory responses. Deramaudt et al. [62] studied the intracellular survival of *S. aureus* in human and mice macrophages treated with SFN. They proposed a mechanism consisting in modulation of p38/JNK signal pathway induced by SFN in macrophages, thus reducing inflammatory response. Additionally, the authors reported that SFN affected *S. aureus* intracellular survival by inducing apoptosis in the bacterium. Then, the combination of both mechanisms supports SFN as a possible treatment for *S. aureus* infection. 

Finally, Belchamber and Donnelly [63] suggested that SFN stimulates phagocytic pathways and improves macrophage phagocytosis of *S. pneumoniae*, *P. aeruginosa* and *H. influenzae* by upregulating Nrf2 in alveolar cells from COPD9 (chronic obstructive pulmonary disease). 

### 2.5. Cancer

**Figure 4:**
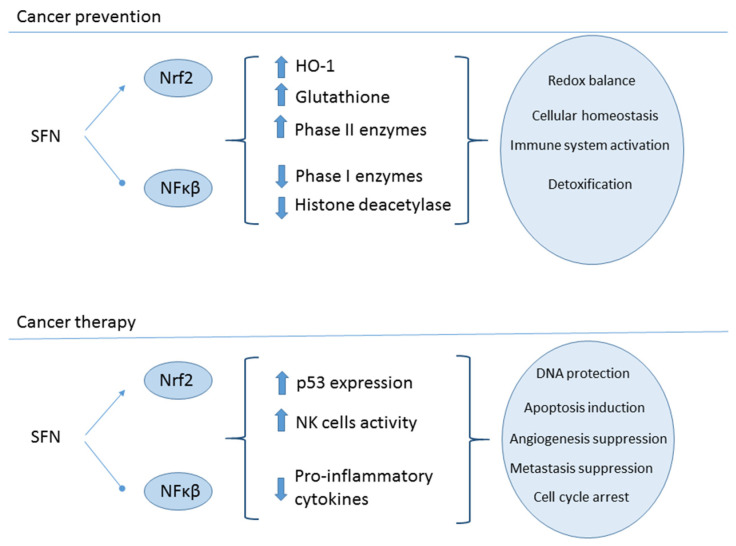
Chemoprotective and chemotherapeutic mechanisms of SFN in cancer cells. SFN exerts chemoprevention by inducing HO-1 and Phase II enzymes, and increasing GSH concentration expression and activating NK cells, as well as downregulating pro-inflammatory cytokines. In both cases, the effect on immune system is mediated by transcription factors Nrf2 and Nfκβ.

Cancer is a multi-factorial disease responsible for around 10 million deaths worldwide per year. The WHO estimates that 30–50% of cancer cases could be prevented. Accordingly, several efforts are made to discover new strategies to fight and most importantly, prevent this disease. SFN is widely recognized as the most potent natural anti-cancer compound. This phytochemical acts at different cancer stages, from development to progression, by exerting a pleiotropic effect. SFN can trigger apoptosis, reducing angiogenesis and metastasis in cancerous cells. At the molecular level, it activates Nrf2, consequently modulating cellular redox homeostasis and stimulating the immune system [64]. Figure 4 shows the mechanism action of SFN as chemoprotective and chemotherapeutic agent. 

Singh et al. [65] studied the effect of administering a SFN analogue on prostate carcinogenesis and pulmonary metastasis in an animal model. The results showed that SFN stimulates NK cells cytotoxicity, thus enhancing immunological function. Also, SFN increased the infiltration of lymphocyte T-cells in prostate tumors resulting in a reduction of metastasis. 

The efficacy of SFN as a possible therapeutic compound has been assayed in different types of cancer cells and tissues. Bessler and Djaldetti [66] investigated the effect of SFN on immunological interaction between PBMC and human colon cancer cell lines. The authors detected a concentration-dependent effect of sulforaphane that inhibited production of pro-inflammatory cytokines in PBMC. SFN acts against colon cancer by different mechanisms: (1) induction of DNA damage in cancerous cells by acetylating the DNA repair protein; (2) activation of pro-apoptotic proteins resulting in induction of apoptosis; (3) activation of Phase II detoxifying proteins through Nrf2; (4) cell cycle arrest by suppressing histone deacetylase inhibitor and telomerase reverse transcriptase [67,68]. Suzuki et al. [69] studied the effect of daily intake of SFN delivered as fresh broccoli sprouts on colon cancer animal model and in humans. Their results indicate that SFN treatment suppressed the formation of aberrant crypt foci and macroscopic tumors in mice and in colon cancer patients. 

Palliyaguru et al. [70] investigated the effect of SFN on breast cancer development in a mouse model exposed to estradiol. The authors found that SFN enhanced cytoprotection by mitigating DNA damage and suppressing lipogenesis. These effects were attributed to activation of Nrf2 by SFN. 

## 3. Clinical Trials Regarding the Effect of Sulforaphane on Immune System

Over the last decade, there are 74 clinical studies that have aimed at evaluating the effect of sulforaphane on different diseases; four of them focused on immune system disorders (www.clinicaltrials.gov). Table 1 shows details about the clinical trials. 

Trial NCT01357070 was designed to test whether consuming a “broccoli smoothie” containing sulforaphane could protect white blood cells from activation in the presence of experimental stress and how long this protective effect would last. To do this, the researchers analyzed inflammatory changes in blood samples taken at different times during the study. The investigators suggest that inducing anti-oxidant enzymes indirectly may be an effective means of providing vascular protection. To date, no results are available.

Trial NCT01183923 hypothesized that since SFN is an inducer of Phase II antioxidant enzymes and broccoli sprouts (BS) are rich in SFN, administration of BS would improve lung and airways function in asthmatic subjects. As a consequence, oxidative stress and inflammation markers would decrease after exposure to allergens. Recruited subjects (n = 1) ingested BS and were exposed to environmental mouse allergen challenge. After, seven daily BS intake markers (nasal epithelial gene expression, urinary oxidative stress biomarkers, serum inflammatory and oxidative stress biomarkers, and basophil activation) were assessed. Unfortunately, this trial was halted because of an adverse event. While no results are available at www.clinicaltrialls.gov, the outcomes of this trial can be found elsewhere [71]. Asthmatic subjects (n = 40) ingested 100 g of BS daily for three days. Effect of SFN was assessed by measuring antioxidant genes expression in nasal epithelial and PBMC, inflammation, and oxidative stress biomarkers, among others. Determinations were conducted before and after BS intake. Since no change in biomarkers and cytoprotective genes expression could be detected, the authors concluded that despite the increase in blood concentration of SFN, BS intake did not improve lung inflammatory response nor antioxidant biomarkers in asthmatic subjects. 

Trial NCT01269723 aimed at evaluating the short-term immunological response to live attenuated influenza virus, and to compare the reaction between smokers and nonsmokers treated with BS (or placebo) homogenate. As response to the treatment, they evaluated the virus charge and inflammation biomarkers (IL6, cytokines, NK cells activation) in nasal mucosa. There are no results available at www.clinicaltrials.gov, however the outcomes of this trial were published in [50]. The main conclusions of this trial was that BS homogenate enhanced immune response against influenza virus, demonstrated by an increase in granzyme B production in peripheral NK cells. 

Trial NCT01845493 consisted of a pilot study about the effect of SFN administration (in the form of BS homogenate) on Nrf2 and Phase II enzymes induction. A total of 16 asthmatic subjects ingested BS homogenate for three days and the outcomes were compared with SFN and placebo controls. Even though the trial ended on 2014, the results are not available so far. 

Trial NCT01845220 aimed at evaluating the effect of SFN (as BS extract) exposure in alcohol-intolerant subjects. After SFN application, subjects (n = 30) were topically exposed to alcohol, and reddened skin area was measured as indicator of SFN protection against irritation. The outcomes indicate that topical application of SFN increased erythema after exposure to alcohol in alcohol-sensitive subjects. 

Trial NCT02885025 studied the effect of administering SFN (as BS extract) to subjects (n = 47) suffering allergic rhinitis to grass. The randomized trial considered a three-week treatment aiming at evaluating the effect of BS extract intake in comparison with administration of corticosteroid (fluticasone) and the combination of both. Before treatment, subjects were exposed (nasal way) to different varieties of grass. The results showed that SFN alone or in combination with fluticasone reduced pro-inflammatory cytokines expression. However SFN exhibited a more limited effect than fluticasone alone.

## 4. Conclusions

Despite the huge amount of information about the effect of SFN on several diseases, especially cancer, research about the effect of SFN on immune system response at molecular and cellular levels is scarce, as well as clinical trials focused on immune system diseases. Sulforaphane exerts a pleiotropic effect on immunological response, and the final effect depends on the cell type. In lymphocyte T-cells, SFN induces ROS production, GSH depletion, and repression of inflammatory cytokines, resulting in suppression of immune and inflammatory responses. This may help in treatment on autoimmune/inflammatory diseases symptoms. In monocytes and macrophages, SFN stimulates immune response by inducing Nrf2, thus triggering antioxidant and anti-inflammatory responses. As a consequence, bacteria survival decreases in infected cells, and virus-infected cells are neutralized by induction of antioxidant enzymes such as HO-1. Additionally, SFN improves immune system, thus helping in prevention and reducing severity of viral pulmonary diseases. In cancer cells, SFN induces apoptosis and cell cycle arrest, as well as antioxidant enzymes that stimulate cellular immune response. Finally, the few clinical trials about the effect of SFN on immune system are not conclusive; this kind of study should be encouraged. 

## Figures and Tables

**Table 1:** Clinical trials regarding the effect of sulforaphane on immune system (www.clinicaltrials.gov).

Clinical Trial Identifier	Title	Study Population	Duration	Sulforaphane (Dose)	Results	Phase	Status
NCT01357070	Effect of Broccoli Sprout on Blood Levels of Sulforaphane to Reduce Responsiveness of Immune System	6 healthy volunteers, London, United Kingdom	34 months (May 2011–January 2014)	broccoli sprout homogenate (70 g dry weight, orally by three consecutive days).	N.I. ^1^	N.A. ^2^	Completed
NCT01183923	Dietary Interventions in Asthma Treatment: Sprouts Study	1 adult, asthmatic, male, white, United States of America	14 months (November 2010–February 2012)	N.I. ^1^ (broccoli sprouts, one serving per day, 7 days, in a sandwich)	N.I. ^1^	N.A. ^2^	Halted
NCT01269723	Effects of Sulforaphane (SFN) on Immune Response to Live Attenuated Influenza Virus in Smokers and Nonsmokers	51 adults, healthy, smokers or nonsmokers, United States of America	28 months (December 2010–March 2013)	N.I.^1^ (broccoli sprouts homogenate)	N.I. ^1^	N.A. ^2^	Completed
NCT01845493	Sulforaphane Supplementation in Atopic Asthmatics (brasma)	16 adults, asthmatics, United States of America	17 months (April 2013–October 2014)	N.I. ^1^ (broccoli sprouts homogenate orally daily, three days)	N.I. ^1^	1	Completed
NCT01845220	Prevention of Alcohol Intolerance	30 adults, older adults, sensitive to alcohol on the skin, Japanese, United States of America	27 months (May 2013–July 2015)	150 nmol of sulforaphane/cm2 of skin in 80% acetone	SFN increased erythema (affected skin area) as response to alcohol exposure	2	Completed
NCT02885025	Effects of Broccoli Sprout Extract on Allergy Rhinitis	47 adults, older adults, allergic rhinitis or healthy	18 months (October 2016–March 2019)	N.I. ^1^ (broccoli sprouts extract)	SFN reduced pro-inflammatory cytokines with and without combination with fluticasone	2	Completed

^1^ Not Informed. ^2^ Not Applicable.
